# Study on the mechanical properties of unloading damaged sandstone under cyclic loading and unloading

**DOI:** 10.1038/s41598-023-33721-y

**Published:** 2023-05-05

**Authors:** Hengbin Zhang, Lehua Wang, Jianlin Li, Huafeng Deng, Xiaoliang Xu

**Affiliations:** 1 Key Laboratory of Geological Hazards on Three Gorges Reservoir Area, Ministry of Education, China Three Gorges University, Yichang City, 443002 Hubei Province China; 2 College of Civil Engineering and Architecture, China Three Gorges University, Yichang City, 443002 Hubei Province China

**Keywords:** Civil engineering, Natural hazards

## Abstract

To reveal the mechanical properties of rocks under stress disturbance and unloading confining pressure, conventional triaxial compression tests, triaxial compression tests on unloading damaged sandstone, and cyclic loading and unloading tests on unloading damaged sandstone were conducted. Then, the evolutionary characteristics of dissipated energy in sandstone under cyclic loading and unloading were explored, and damage variables were proposed. The crack development characteristics were analyzed from a microscopic perspective. The study results reveal that: (1) the sandstone exhibits obvious brittle failure under different stress paths, and the macroscopic failure mode is dominated by shear failure. As the number of cycles increases, the load-bearing capacity, elastic modulus, and deformation modulus of the sandstone will be significantly reduced if it suffers greater unloading damage. (2) The cyclic action in the early stage inhibits the development of the internal fracture. However, the inhibitory effect is significantly reduced for specimens with larger unloading quantities. The damage variable in the cyclic loading and unloading is about 50.00% of that in the unloading, indicating that unloading confining pressure is the dominant factor for specimen failure. (3) The extension of microcracks within the sandstone is dominated by intergranular cracks, and the number of cracks increases with the increase of unloading quantity. After cyclic loading and unloading, the structure becomes looser. The test results deepen the understanding of rock mechanical behavior and fracture evolution under cyclic loading and can provide a basis for structural stability improvement under stress disturbance and unloading confining pressure.

## Introduction

The mechanical nature of engineering excavation activities is stress redistribution in the rock mass, inducing stress unloading in one or more directions. Therefore, any engineering excavation will result in certain degrees of unloading damage. Most traditional studies of rock mechanical behavior focus on monotonic loading and unloading conditions of intact rocks, while actual rock projects are often subjected to a combination of unloading, cyclic loading and unloading during construction and service.During the excavation of high slopes, mine tunnels, traffic roads and tunnels, as well as in the operation of dams and other projects, the rock masses after excavation and unloading are affected by cyclic load disturbance to varying degrees. The difference between their deformation characteristics and traditional mechanical behavior has been a hot research topic in recent years. Thus, an in-depth understanding of the cyclic loading and unloading characteristics of unloading damaged rocks is important for engineering stability analysis and safety evaluation^1,2^.

In recent years, global scholars have studied the mechanical properties and wave velocity of rocks under cyclic disturbance^3,4^. Zhu et al.^5^ and Zhou et al.^6^ performed cyclic dynamic loading tests on specimens with holes and defects and concluded that the defect area ratio significantly influenced the crack expansion pattern. Li et al.^7^ examined the acoustic emission of six types of rocks, namely, granodiorite, hornfels, skarn, copper ore, wolfram-molybdenum ore and lead–zinc ore, during cyclic loading and unloading and concluded that rock failure could be predicted with the "relatively tranquil period" as a reference basis. Based on a multi-field coupler and ultrasonic test system, Zhang et al.^8^ tested the longitudinal and transverse wave velocities of specimens during unloading and loading. They concluded that transverse waves are approximately linear, and longitudinal waves undergo three stages: continuous growth, oscillation, and rapid decay. Moreover, many scholars have considered the effect of cyclic loading in true triaxial physical tests. Gao et al.^9^ and Feng et al.^10^ conducted true triaxial cyclic loading and unloading tests to analyze the evolution of residual deformation of granite, sandstone and barite during the cycle. Based on their research results, Duan et al.^11^ explored the effect of intermediate principal stress of tiered cycles on coal rock deformation during the cycle. At present, more and more scholars analyze the damage evolution of specimens from the energy perspective. Zong et al.^12^ studied the characteristics of the stress–strain curves and strength evolution laws through the re-loading tests of samples damaged to various degrees under different stress states. Xu et al.^13^ and Cai et al.^14^ explored the cumulative change law of damage in mudstone and marble under tiered cyclic loading and unloading from the energy perspective and derived the response relationship between plastic strain and energy density for each cycle. Yang et al.^15^ conducted a triaxial cyclic unloading test on sandstone and introduced the "energy dissipation ratio", whose variation was divided into five stages: linear decrease, stable development, slow increase, sudden increase, and gentle change through the full stress–strain curve. Gao et al.^16^ conducted uniaxial loading–unloading experiments on five types of rocks and investigated their energy evolution characteristics, and identified the damage and crack propagation thresholds. Liu et al.^17^ and Cheng^18^ normalized the dissipated energy and used it to characterize rock damage. Li et al.^19^ and Gao et al.^20^obtained the evolution law of input energy, elastic energy, and damage energy during uniaxial cyclic loading and unloading of sandstone, and characterized the damage evolution of sandstone by damage energy. Gong et al.^21^ proposed a new energy criterion, the residual elastic energy index, through uniaxial cyclic loading and unloading tests.

According to the current research results, scholars have analyzed the mechanical properties, failure modes and damage evolution laws of rocks under cyclic loading from multiple aspects. Some even proposed intrinsic structure models and obtained valuable research results, providing an important research idea for the present paper. However, previous studies mainly focus on intact rocks, and few scholars have analyzed the mechanical properties of unloading damaged rocks under cyclic loading and unloading. It is necessary to comprehensively consider the effect law of unloading as well as cyclic loading and unloading on the mechanical properties of rocks.

In this paper, considering the impact of cyclic disturbance after excavation and unloading of actual projects, conventional triaxial compression tests, unloading tests, and cyclic loading and unloading tests were conducted on sandstone to study the differences in mechanical parameters, deformation parameters and failure modes. The evolution law of dissipated energy in the unloading process and cyclic loading and unloading process was explored for comparative analysis, and damage variables were proposed based on the evolution characteristics of dissipated energy. Additionally, the development characteristics of cracks were analyzed from a microscopic perspective.

## Experimental program design

### Sample preparation

**Figure 1:**
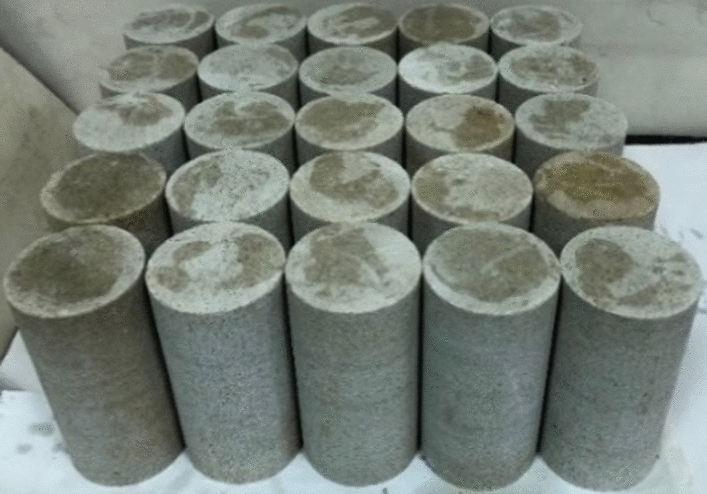
Complete samples.

**Figure 2:**
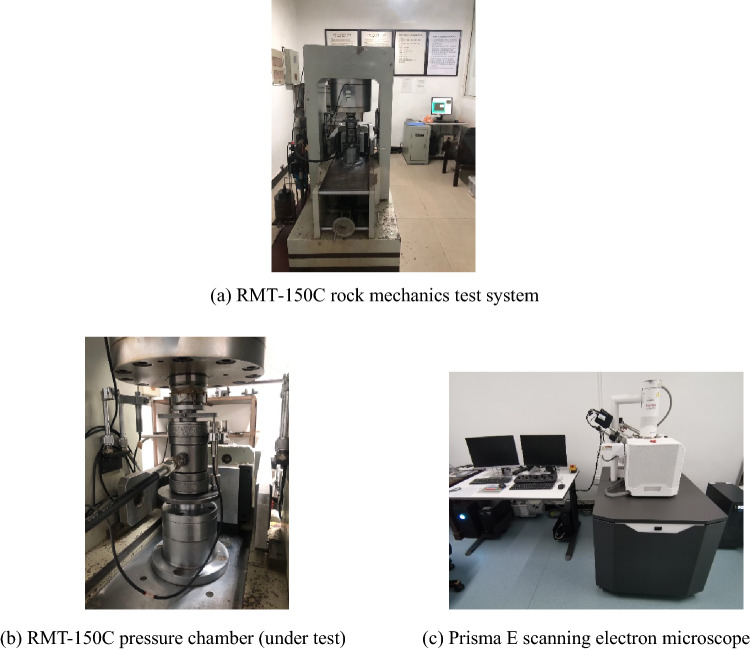
Test instruments.

Typical sandstone in the Three Gorges reservoir area was selected as the research object. After retrieving from the site, the rock samples were drilled and made into standard specimens of 50 mm in diameter and 100 mm in height according to the specification. The wave velocity of the specimen was measured, and those with large differences were excluded to reduce the dispersion. The test specimens are shown in Fig. 1. The RMT-150C rock mechanics test system and the Prisma E scanning electron microscope are shown in Fig. 2.

### Test methodology

**Figure 3:**
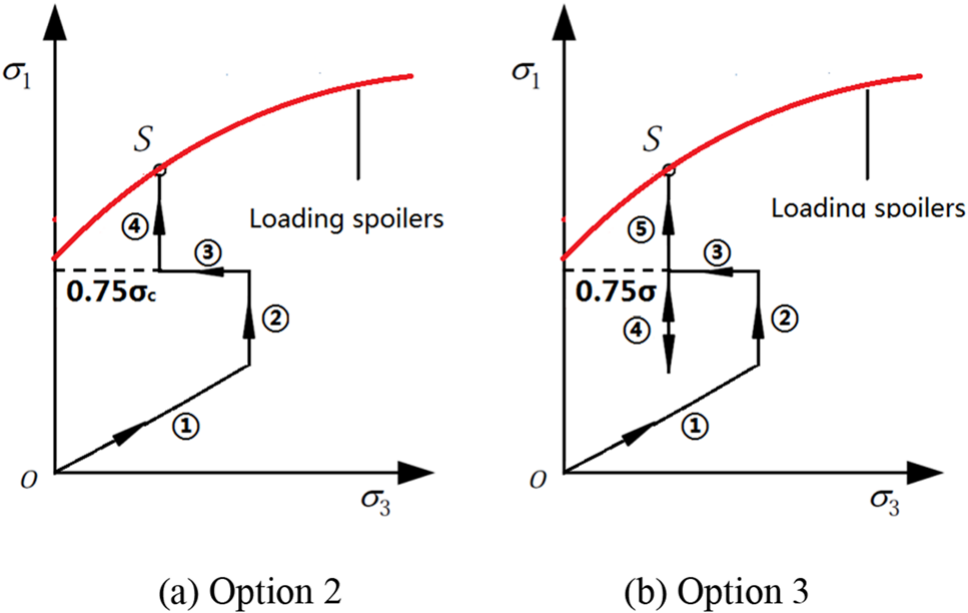
Stress path.

The test program consists of three options, and each was repeated three times to reduce errors. Option 1: conventional triaxial compression test. Option 2: triaxial compression test for unloading damaged samples (as shown in Fig. 3a: ① raise confining pressure to hydrostatic pressure. ② Lift axial pressure to the initial axial stress level. ③ Unload confining pressure to different unloading quantities. ④ Increase axial pressure until specimen failure). Option 3: cyclic loading and unloading test on unloading damaged samples (as shown in Fig. 3b: ① lift confining pressure to hydrostatic pressure. ② Raise axial pressure to the initial axial stress level. ③ Unload confining pressure to different unloading quantities. ④ Conduct cyclic loading and unloading in the axial pressure direction. ⑤ Increase axial pressure until specimen failure).

Option 1 is the conventional triaxial compression test which aims to obtain the peak strength at eight different confining pressures (12.5, 13.0, 13.5, 14.0, 14.5, 15.0, 17.0, and 20.0 MPa) and determine the design value of axial pressure (75% of the peak strength) for Options 2 and 3.

Option 2 is a triaxial compression test considering different initial unloading damaged specimens, with 75% of the peak strength at the enclosure pressure of 20.0 MPa in Option 1 as the initial axial stress level. The unloading enclosure pressure was unloaded to different unloading quantities. Based on the local geographical conditions and the test schemes in previous studies^22,23^, eight unloading quantities were selected: 100% (corresponding to a confining pressure of 12.5 MPa), 93.33%, 86.67%, 80.00%, 73.33%, 66.67%, 40.00%, and 0% (corresponding to a confining pressure of 20.0 MPa). Among 86.67%, 80.00%, 73.33%, 66.67%, 40.00%, and 0% (corresponding to a confining pressure of 20.0 MPa), their corresponding confining pressure of the unloading end-point was the same as that in Option 1, with an unloading rate of 0.05 MPa/s, which caused different degrees of initial unloading damages to specimens before loading the axial pressure until failure occurred.

In Option 3, the confining pressure was kept unchanged after the unloading test in Option 2 and the axial pressure was gradually unloaded to 20.0 MPa. Then the axial pressure was loaded to 75% of the peak strength (the rate of loading and unloading was 1 kN/s), and the loading and unloading cycle was repeated five times. Then, the axial pressure was increased until the specimen failed.

## Results and analysis

### Analysis of strength parameters

**Figure 4:**
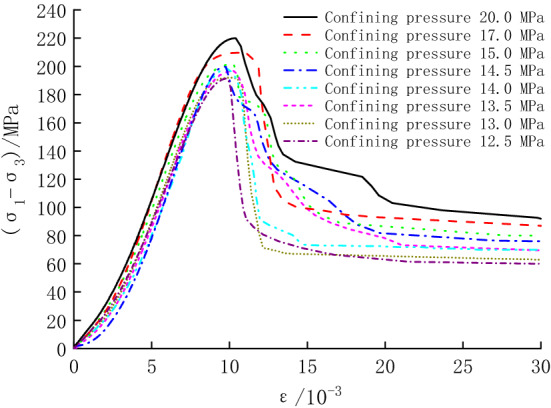
Stress–strain curves for specimens in Option 1.

Figure 4 shows the stress–strain curves of the conventional triaxial compression test for the specimens in Option 1. According to Fig. 4, the peak strength of the specimens is listed in Table 1. It can be seen that with the increase in axial compression, stress drop immediately occurs after specimens reach peak strength. The axial peak strain appears within 1.06–1.22%, which is less than 3%, and the breakage belongs to brittle failure^22^. As can be seen in Fig. 4, the peak strength gradually increases with the increasing confining pressure. The elastic modulus is 32.12–34.41 GPa, which indicates that the dispersion of the specimens is small.

**Table 1:** Peak strength (deviatoric stress).

Confining pressure/MPa	Unloading quantity (%)	Peak strength/MPa		
		Option 1	Option 2	Option 3
12.5	100	191.28	167.12	167.12
13.0	93.33	192.03	172.11	169.97
13.5	86.67	196.78	179.08	177.60
14.0	80.00	197.22	190.22	183.90
14.5	73.33	199.95	192.13	186.72
15.0	66.67	201.16	194.54	189.57
17.0	40.00	209.62	204.72	199.88
20.0	0	219.96	219.96	210.92

**Figure 5:**
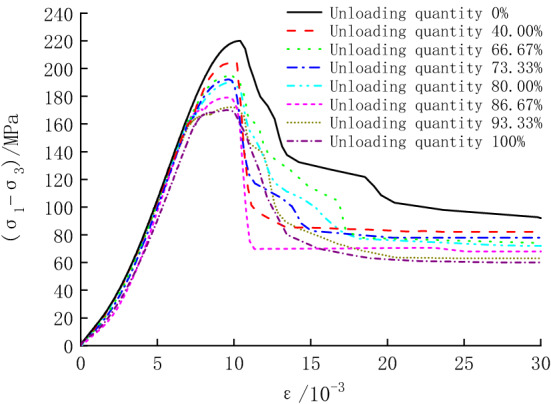
Stress–strain curves for specimens in Option 2.

The obtained stress–strain curves in Option 2 are shown in Fig. 5. The peak strength of the specimens can be obtained in Fig. 5, and some parameters are shown in Table 1. It can be seen from Table 1 that the overall changing pattern of the peak strengths remain the same, but the decay becomes more prominent with the increasing unloading quantity.

**Figure 6:**
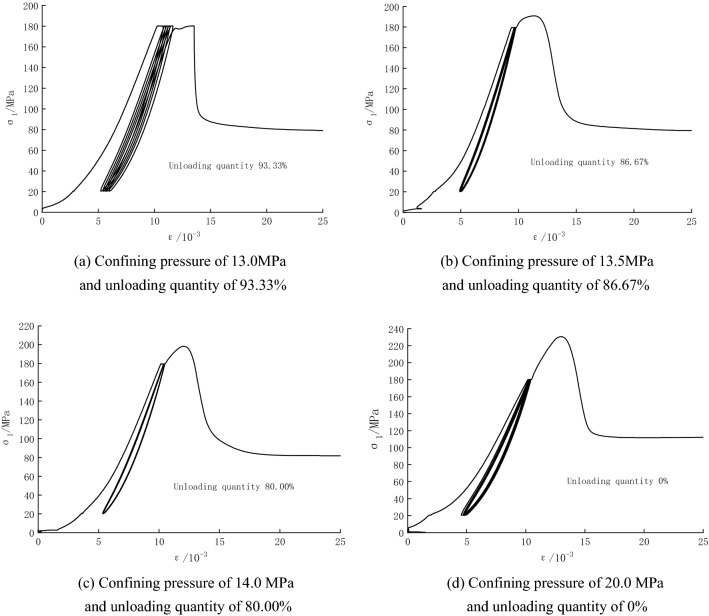
Stress–strain curves for some specimens in Option 3.

Stress–strain curves for some specimens in Option 3 are shown in Fig. 6 (only some test curves are listed due to space limitation). It can be seen that the axial strain during cyclic loading and unloading increases gradually as the unloading quantity increases, and the stress–strain curves of the specimens during cyclic loading and unloading are closely spaced, generating five hysteresis loops. Except for the hysteresis loop curve at the confining pressure of 13.0 MPa (unloading quantity of 93.33%), which shows a sparse dense sparse trend, the hysteresis loops at other confining pressure show a sparse dense trend^24,25^ (obtained by combining the stress–strain curves with the computational analysis results in the later section). Additionally, the specimens under cyclic loading and unloading still have obvious stress drop segments, and the breakage belongs to brittle failure.

Based on Fig. 6, the peak strength of the specimens is listed in Table 1. It can be seen that under cyclic loading and unloading, the trend of the peak strength of unloading damaged specimens with the confining pressure is similar to that in Option 2. However, with the increase in the confining pressure, the growth of peak strength gradually slows down, and the axial peak strain gradually decreases, with a minimum value of 167.12 MPa and a maximum value of 210.92 MPa.

**Figure 7:**
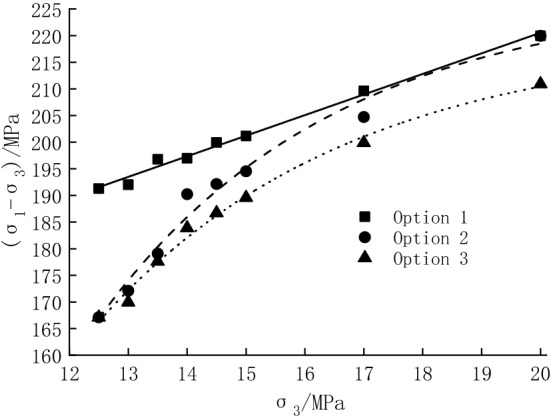
Peak strength comparison.

Based on the test data in Table 1, a peak strength comparison graph of the three tests was plotted (Fig. 7). At the confining pressure of 12.5 MPa, the unloading quantity is 100%, and the specimen fails without cyclic loading and unloading; during unloading, the peak strength of the specimen is reduced by 24.16 MPa (12.63%). At the confining pressure of 20.0 MPa, the unloading quantity is 0, and the strength loss of the specimen is attributed to cyclic loading and unloading, which is 9.04 MPa (4.11%). At the unloading quantity of 40.00–93.33%, the peak strength is reduced by 4.90–19.92 MPa (2.34–10.37%) under unloading action. After superimposing cyclic loading, the peak strength is reduced by 9.74–22.06 MPa (4.65–11.49%). It can be concluded that cyclic loading and unloading can further weaken the bearing capacity of specimens. When unloading is combined with cyclic loading and unloading, the unloading action is the main reason for the decrease in the peak strength of specimens.

Overall, the peak strength of specimens is ranked as Option 1 > Option 2 > Option 3. The reason is that the unloading process induces more new cracks than the conventional triaxial compression process, while the cyclic loading and unloading process intensifies the extension of internal cracks and the damage to the specimen^2^. The result indicates that the cumulative damage under cyclic unloading and loading significantly affects the mechanical properties of rocks.

### Analysis of deformation characteristics

**Figure 8:**
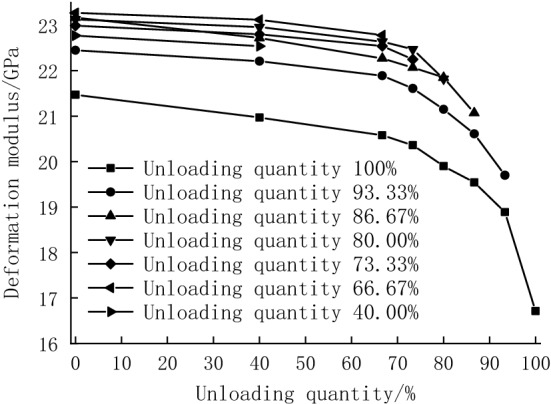
The curve of deformation modulus and unloading quantity at the unloading stage.

The variation in deformation modulus during unloading (the difference in deformation modulus between the starting and end point of unloading) in Option 2 is given in Table 2. A schematic diagram of the variation in deformation modulus during the unloading stage is drawn in Fig. 8 based on Table 2. It can be seen that the deformation modulus gradually decreases with the increasing unloading quantity, and the decrease is first steady and then becomes steep. When the unloading quantity is 40.00%, 66.67%, and 73.33%, the deformation modulus decreases by 1.00%, 2.13%, and 3.22% during unloading, with an average of 2.12%, indicating ageneral slight decrease. When the unloading quantity is 80.00%, 86.67%, 93.33%, and 100%, the deformation modulus decreases dramatically by 5.69%, 9.05%, 12.24%, and 22.17%, with a mean change of 12.29%. In the unloading stage, the decrease of the deformation modulus with the increasing unloading quantity is more obvious.

**Table 2:** Deformation modulus of Option 2.

Confining pressure/MPa	Unloading quantity (%)	0%	40.00%	66.67%	73.33%	80.00%	86.67%	93.33%	100%	Change in deformation modulus	
12.5	100	21.47	20.97	20.58	20.36	19.90	19.54	18.89	16.71	4.76	22.17%
13.0	93.33	22.45	22.21	21.89	21.61	21.15	20.61	19.70	–	2.75	12.24%
13.5	86.67	23.17	22.72	22.27	22.07	21.85	21.07	–	–	2.10	9.05%
14.0	80	23.13	22.96	22.64	22.47	21.82	–	–	–	1.32	5.69%
14.5	73.33	22.99	22.80	22.54	22.25	–	–	–	–	0.74	3.22%
15.0	66.67	23.27	23.12	22.78	–	–	–	–	–	0.50	2.13%
17.0	40	22.77	22.54	–	–	–	–	–	–	0.23	1.00%

In Option 3, the elastic modulus was calculated from the average unloading elastic modulus, and the unloading elastic modulus *E*_*ur*_^26^ was obtained from the ratio of axial stress to axial strain during cyclic loading and unloading as the slope of two intersections of the hysteresis loop.

1 $$E_{ur} = \frac{{\Delta \left( {\sigma_{1} - \sigma_{3} } \right)}}{{\Delta \varepsilon_{1} }}$$

where $\Delta \left( {\sigma_{1} - \sigma_{3} } \right)$ is the increment of axial stress at the two end points of the hysteresis loop; $\Delta \varepsilon_{1}$ is the increment of axial strain at the two end points of the hysteresis loop.

**Figure 9:**
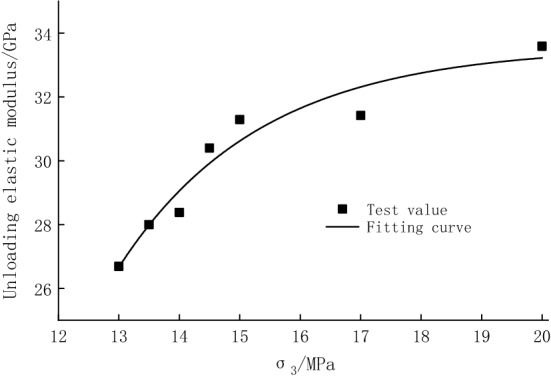
Relationships between unloading elastic modulus and confining pressure.

The unloading elastic modulus was calculated according to Eq. (1) and shown in Table 3, and the relationship curve between the unloading elastic modulus and the confining pressure was plotted (Fig. 9). It can be seen that the unloading elastic modulus increases from 26.69 to 33.59 GPa as the confining pressure increases (the unloading quantity decreases), and then gradually levels off. In the single test, the elastic modulus showed a trend of first increasing and then decreasing with the increased number of cycles, which indicated that the development of internal cracks in the specimen could be suppressed at the beginning of the cycle. However, at the later stage of the cycle, the internal cracks gradually developed, and new cracks were generated. Thus, the elastic modulus slightly decreased. As the confining pressure decreases (the unloading quantity increases), the peak appears earlier. The peaks appear in the 4th cycle when the confining pressure decreases from 20.0 to 14.0 MPa (the unloading quantity increases from 0 to 80.00%), and the values are 33.66 GPa, 31.47 GPa, 31.34 GPa, 30.62 GPa, and 28.63 GPa, respectively. With the further decrease of the confining pressure to 13.0 MPa (the unloading quantity increases to 93.33%), the peaks of 28.34 GPa and 26.77 GPa occur in the 3rd cycle.

**Table 3:** Unloading elastic modulus of each hysteresis loop.

Confining pressure/MPa	Unloading quantity (%)	Unloading elastic modulus/GPa					
		Cycle 1	Cycle 2	Cycle 3	Cycle 4	Cycle 5	Average value
13.0	93.33	26.74	26.74	26.77	26.72	26.47	26.69
13.5	86.67	28.23	28.19	28.34	27.89	27.35	28.00
14.0	80.00	27.81	28.36	28.50	28.63	28.59	28.38
14.5	73.33	30.10	30.32	30.42	30.62	30.52	30.40
15.0	66.67	31.15	31.28	31.32	31.34	31.32	31.29
17.0	40.00	31.33	31.43	31.43	31.47	31.43	31.42
20.0	0	33.51	33.57	33.60	33.66	33.63	33.59

Under cyclic loading and unloading, the specimen damage intensifies with the increasing number of cycles, which leads to a simultaneous increase of strain. Due to the poor inhibitory effect of low confining pressure on the deformation of the specimen, the bearing capacity of the specimens under low confining pressure is significantly reduced. In contrast, high confining pressure has a strong strain suppression ability, so the specimens under high confining pressure still have a high bearing capacity.

**Figure 10:**
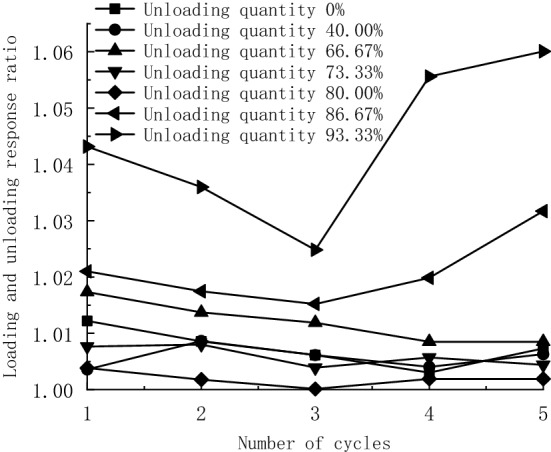
Schematic diagram of loading and unloading response ratio.

The loading and unloading response ratio theory is vital in predicting accidents such as structural failure of the rock. Therefore, the loading and unloading response ratio *Y* is used to characterize the whole cyclic unloading process quantitatively and is calculated according to Eq. (2)^27,28^ (Table 4). Additionally, a schematic diagram of the loading and unloading response ratio is drawn (Fig. 10).

2 $$y = \frac{{E_{ - } }}{{E_{ + } }}$$

where *E*_+_ and *E*_*−*_ are the elastic modulus of the loaded and unloaded sections, respectively.

**Table 4:** Statistical table of loading and unloading response ratio.

Confining pressure/MPa	Unloading quantity (%)	Loading and unloading response ratio				
		Cycle 1	Cycle 2	Cycle 3	Cycle 4	Cycle 5
13.0	93.33	1.043	1.036	1.025	1.056	1.060
13.5	86.67	1.021	1.017	1.015	1.020	1.032
14.0	80.00	1.004	1.002	1.000	1.002	1.002
14.5	73.33	1.008	1.008	1.004	1.006	1.004
15.0	66.67	1.017	1.014	1.012	1.009	1.009
17.0	40.00	1.004	1.009	1.006	1.004	1.006
20.0	0	1.012	1.009	1.006	1.003	1.007

As shown in Fig. 10, when the unloading quantity is less than 80.00%, the response ratio fluctuates under the effect of five loading and unloading cycles. However, the fluctuation is not large, and no abnormal abrupt change occurs. After the unloading quantity exceeds 80.00%, the response ratio decreases and then increases as the number of cycles increases. The loading and unloading response ratio of specimens with unloading quantities of 80.00% and 93.33% decrease by 0.006 and 0.018 in the 1st–3rd cycles, and increase by 0.017 and 0.035 in the 3rd–5th cycles.

Internal cracks are generated during the unloading stage and develop more fully as the unloading quantity increases. The above results show that 80.00% unloading quantity is a critical point. The internal cracks are not fully developed when the unloading quantity is less than 80.00%. Thus, the specimen still has a high bearing capacity in the subsequent loading and unloading cycle, which is the same as the conclusion of the previous elastic modulus analysis. When the unloading quantity exceeds 80.00%, a large number of cracks are generated inside the specimen. At the beginning of cyclic loading and unloading, the pores and initial cracks are gradually compacted, the specimens tend to be stable and the loading and unloading response ratio decreases. As the number of cycles increases, the microcracks and fissures inside the specimens gradually develop, and the overall rock sample is close to a failure state. Therefore, the loading and unloading response ratio increases steeply, and rock failure is more likely to occur in the subsequent repeated loading process. It can be seen that the loading and unloading response ratio of the rock sample significantly increases before failure, which is consistent with the previous test law^29,30^.

**Figure 11:**
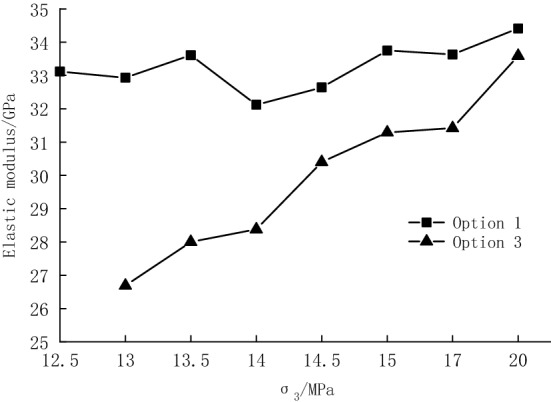
Elastic modulus comparison.

The elastic modulus of Option 1 and Option 3 is plotted in Fig. 11. It can be seen that in Option 3, the hysteresis loop shows an outward convex shape, so its elastic modulus is generally smaller than that obtained from conventional triaxial compression tests at the corresponding confining pressure. During cyclic loading and unloading, the accumulated damage of the specimens increases with the increasing number of cycles; the elastic modulus of the specimens becomes smaller and further decreases due to the weak inhibition of the ring strain by the low confining pressure (high unloading quantity). These results are consistent with the experimental data in the previous paper. The difference in the elastic modulus is small at high confining pressure (low unloading quantities), which indicates that high circumferential pressure has a certain inhibitory effect on the development of internal cracks. Different from intact specimens, unloading damaged specimens undergo the closure and expansion of the primary fractures, generation of new fractures, and aggravation of damage after cyclic loading and unloading, indicating that the unloading process and the cyclic loading and unloading process significantly affect the mechanical properties of the rock. The unloading quantity corresponding to the confining pressure of 12.5 MPa is 100%, and the specimen fails directly due to unloading, so the unloading quantity of 100% is not considered.

**Figure 12:**
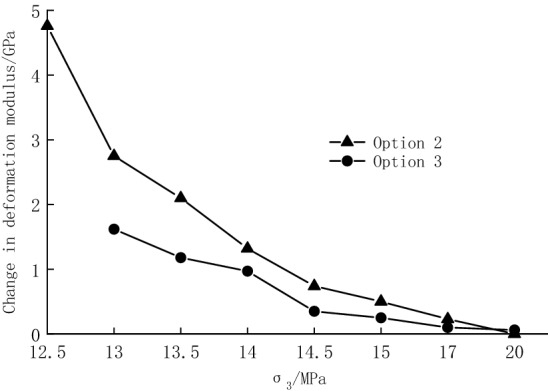
Comparison of changes in modulus of deformation.

The deformation modulus changes in the unloading phase in Option 2 and the cyclic loading and unloading phase in Option 3 are plotted in Fig. 12. It is found that the deformation modulus changes in the cyclic loading and unloading phase are generally less dramatic than those in the unloading phase at the same surrounding pressure. The difference in the deformation modulus change gradually increases from 0.13 to 1.13 GPa as the unloading quantity increases from 0 to 93.33%, indicating that the unloading process causes more deformation than the cyclic loading and unloading process. A similar pattern can be obtained from the strain change discussed in the previous section. When the confining pressure is small (the unloading quantity is large), the ability of the specimens to resist internal cracks is poor, and the deformation of the specimens caused by unloading is large. Although the cracks inside the specimen are closed at the beginning of cyclic loading and unloading, they will develop as the number of cycles increases, leading to a decrease in the deformation modulus and an increase in the variation. On the contrary, under a larger confining pressure (smaller unloading quantity), the specimens have a stronger capacity to resist cracks, and the initial cracks inside the specimens are less likely to develop. Although the cyclic loading and unloading causes damage to the specimens, the specimens still have a strong bearing capacity. Thus, the deformation modulus decreases slightly, with little change. The specimen with 12.5 MPa confining pressure exhibits unloading failure, and there is no cyclic loading and unloading process in the test, so its effect on the specimen is not discussed.

### Analysis of failure characteristics

**Figure 13:**
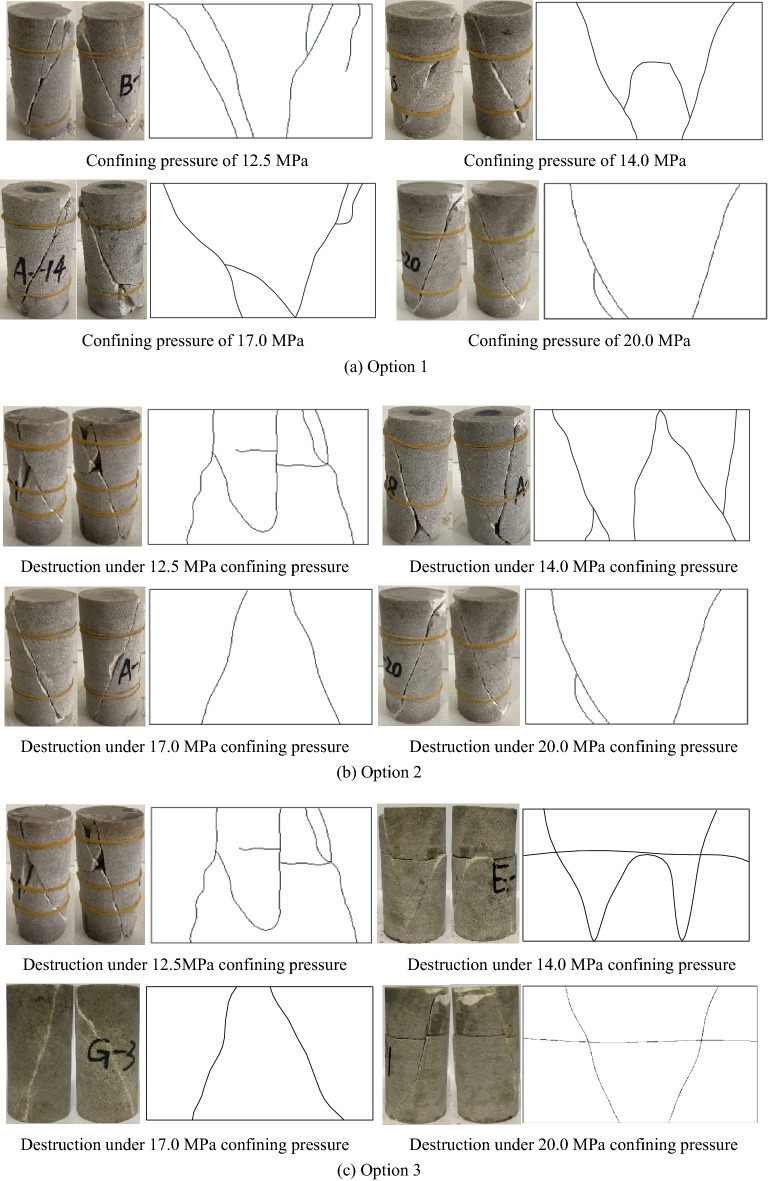
Schematic diagram and sketch of typical rock sample fracture.

Due to the space limitation, only the macroscopic failure characteristics of some specimens are listed in Fig. 13. From the figure, it can be seen that.

1. In the conventional triaxial compression test in Option 1, the macroscopic failure mode of the sandstone is dominated by shear failure. At the confining pressure of 12.5 MPa, in addition to generating a penetrating shear cracks, a small number of secondary cracks are also generated, and the failure is serious. At the confining pressure of 20 MPa, there is only one shear crack through the sandstone, forming a wedge shape of 45°–65°.
2. In the triaxial compression test of the unloading damaged specimen in Option 2, the failure of the specimen is most serious at the confining pressure of 12.5 MPa (100% unloading quantity) since crack formation and development are less likely to be inhibited under low confining pressure. Macroscopically, most failure is tensile-shear failure, and secondary tensile cracks are formed on both sides of the shear surface. In the test of unloading damaged rock samples, the rock samples suddenly lose strength, especially when the unloading quantity is large, and a crisp rupture sound can be heard, which also indicates that the sample undergoes intense brittle failure. With the decrease in the unloading quantity, the tensile crack at the end of the specimen gradually decreases, and the failure mode gradually changes from tensile-shear failure to compression-shear failure. The macroscopic failure surface is a single shear failure surface, the failure mode at this time is the same as that in the conventional triaxial compression test.
3. In the cyclic loading and unloading test of unloading damaged specimens in Option 3, specimen failure is most serious at the confining pressure of 13.0 MPa (unloading quantity of 93.33%). However, complete penetrating shear cracks are still predominant, with a small number of secondary cracks. With the increase of the confining pressure (decrease of the unloading quantity), the secondary cracks gradually disappear, forming a single penetrating shear surface. Option 3 is usually accompanied by the generation of one or more transverse cracks, which is due to the reduction of the axial stress. At this point, the confining pressure remains constant, and the bias stress on the specimen decreases, generating tensile stress in the horizontal plane in the vertical direction and "pulling off" the specimen, similar to the phenomenon in the previous paper^31^.
4. At the initial stage of cyclic loading and unloading, the fracture-damaged rock particles fill into nearby fractures. The fractures and particles are continuously embedded into a more dense state. However, in the later stage of cyclic loading, cracks are first formed in the middle weak surface, and transverse cracks are eventually formed, with fragments, a large number of debris and powder particles accompanied by local shear slip. The reason is that the repeated friction of the internal structural surface under cyclic loading destroys the original internal structure, and the adhesion between particles is weakened, causing more particle debris on the original shear rupture surface^31^.

### Analysis of damage variables based on dissipated energy density

**Figure 14:**
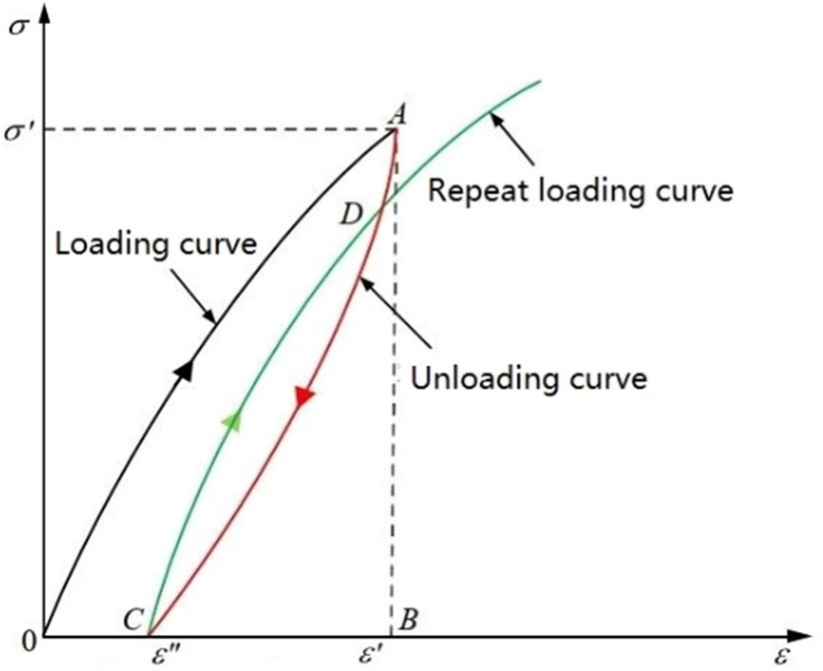
Schematic diagram of energy density calculation for rock samples.

The elastic energy stored in the rock is reversible, and the area of the hysteresis loop reflects the amount of the dissipated energy. According to the basic characteristics of the stress–strain curve, the elastic energy density and dissipated energy density of the specimen in the unloading phase and the cyclic loading and unloading phase can be calculated, as shown in Fig. 14. The area under the loading curve in the stress–strain curve indicates the total energy density *U* (area enclosed by OAB) absorbed by the specimen, which is the work done by the test apparatus on the specimen. The area under the unloading curve represents the elastic energy density *U*^*e*^ stored in the specimen (area enclosed by ABC). The accumulated damage inside the specimen is determined by the dissipated energy density *U*^*d*^ (area enclosed by OAC), which is calculated by subtracting *U*^*e*^ from *U*, and the area between the loading and unloading curves. The specific calculation expression^32^ is:

3 $$\begin{gathered} U_{i}^{e} = \int_{{\varepsilon^{\prime\prime}}}^{{\varepsilon^{\prime}}} {\sigma_{i} d\varepsilon_{i} } \hfill \\ U_{i}^{d} = \int_{0}^{{\varepsilon^{\prime}}} {\sigma_{i} d\varepsilon_{1} } - \int_{{\varepsilon^{\prime\prime}}}^{{\varepsilon^{\prime}}} {\sigma_{i} d\varepsilon_{i} } \hfill \\ \end{gathered}$$

where $\sigma^{\prime}$ is the stress at a point on the curve, $\varepsilon^{\prime}$ is the corresponding strain, and $\varepsilon^{\prime\prime}$ is the corresponding strain when $\sigma^{\prime}$ is unloaded.

Since there is unloading axial pressure in Option 3, only the specimens in Option 3 were analyzed in terms of energy density. Additionally, since the lower limit of cyclic loading and unloading in Option 3 is 20.0 MPa, only the curve above σ_1_ = 20.0 MPa was analyzed for the energy density at the unloading phase and the cyclic loading and unloading phase. The energy density calculation for the 5th cycle requires the 6th cycle curve (which was not performed in the test), so only the energy density of the first four cycles was calculated, as shown in Table 5.

**Table 5:** Statistical table of the energy density of each hysteresis loop (Unit: kJ/m^3^).

Confining pressure/MPa	Cycle 1		Cycle 2		Cycle 3		Cycle 4	
	Ud1	U1	Ud2	U2	Ud3	U3	Ud4	U4
13.0	110.29	453.82	108.52	453.02	107.24	451.10	123.25	470.34
13.5	96.01	441.12	87.44	432.55	80.01	423.40	82.53	426.56
14.0	68.52	395.42	63.63	390.66	61.27	388.41	59.97	387.18
14.5	67.13	393.07	61.00	386.89	59.05	384.68	57.20	382.07
15.0	64.23	385.70	60.82	381.79	58.34	379.33	56.63	377.42
17.0	58.44	365.39	54.86	362.58	52.77	362.25	52.10	360.95
20.0	52.04	340.41	49.77	338.43	47.41	337.13	46.12	335.90

According to the data shown in Table 5, it can be found that:

1. With the increasing number of cycles, the dissipated energy density is significantly higher than that corresponding to low unloading quantity when the unloading quantity is greater than 80.00% and shows a trend of first decreasing and then increasing. This result indicates that the internal crack of the specimen is more fully developed under the unloading effect, and the initial cycling plus the unloading effect can delay but cannot prevent crack development. The 3rd cycle result is the lowest (80.01 kJ/m^3^ and 107.24 kJ/m^3^). The dissipated energy density gradually increases in the subsequent cycles, showing that the low confining pressure has a weak ability to inhibit fracture development. When the unloading quantity is less than 80.00%, the dissipated energy density gradually decreases, and the overall decrease is insignificant. The phenomenon indicates that a small number of cracks are formed in the specimen under the unloading effect in the early stage, but the cracks gradually close under the initial cyclic loading and unloading effect and high confining pressure. With the increasing number of cycles, the internal damage of the specimen gradually increases, and the closed cracks gradually develop, accompanied by new cracks, which eventually reduces the bearing capacity of the specimen. According to the results of previous studies, it can be predicted that the dissipated energy density of the 5th cycle should be higher than that of the 4th test.
2. As the unloading quantity increases (the confining pressure decreases), the dissipated energy density first increases slowly and then increases steeply, and the inflection point is at 80.00% of the unloading quantity (confining pressure of 14.0 MPa). The increments of the slow increase section and steep increasse section are about 14.51 kJ/m^3^ and 49.03 kJ/m^3^, which indicates that the internal damage of the specimen with high unloading quantity is relatively serious. Additionally, the sharp increase in dissipated energy can lead to a dramatic decrease in the energy storage performance and a sharp increase in the deformation of the specimen (as also found in the previous analysis), and the stable structure gradually develops into a rupture structure. This result indicates that as the confining pressure increases (the unloading quantity decreases), the dissipated energy density decreases. The increase in confining pressure increases the intensity of energy input and improves the efficiency of energy accumulation while inhibiting energy dissipation and release due to the rupture or destruction of rock samples. The unloading process increases the ratio of dissipated energy to the absorbed energy of the rock sample and reduces the released elastic strain energy, with significant energy dissipation. Therefore, during repeated loading, the specimen is more likely to fail, and its bearing capacity is significantly reduced.

The damage of sandstone specimens is a process in which small internal natural cracks and fissures are continuously compacted under pressure and gradually expand into macroscopic cracks to generate new cracks. The evolution of damage variables can be seen as an irreversible, energy-consuming process of the internal structure of the material, which reflects the change law of the rock from the compaction of fractures and generation of new fractures to destruction from another perspective. The damage variables defined according to previous equations^33,34^ cannot be integrated considering the unloading process and cyclic loading and unloading process. Therefore, the damage variables are re-defined, as shown in Eq. (4).

4 $$D_{i} = \frac{{U_{0}^{d} + \sum\limits_{i = 1}^{N} {U_{i}^{d} } }}{{U_{0} + \sum\limits_{i = 1}^{N} {U_{i} } }}$$

where: $D$ is the damage variable, $U_{0}^{d}$ is the dissipated energy density in the unloading section, $U_{0}$ is the total energy density in the unloading section, $U_{i}^{d}$ is the dissipated energy density in the i-th cycle, and $U_{i}$ is the total energy density in the i-th cycle.

**Figure 15:**
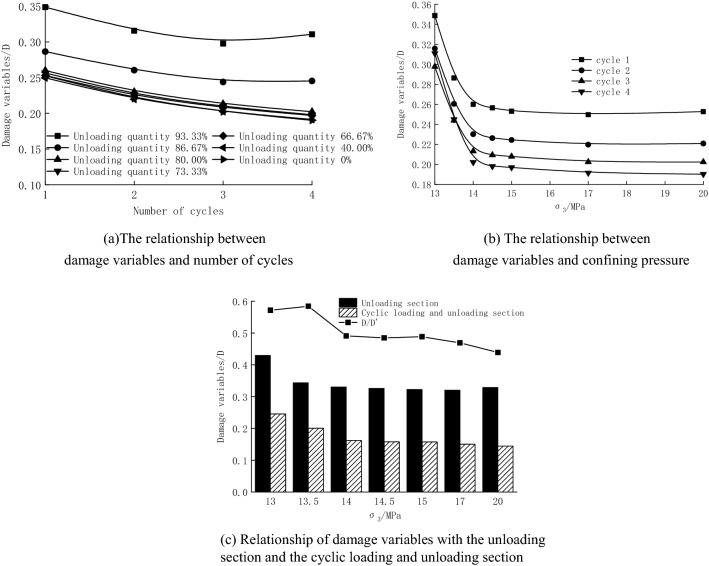
Schematic diagram of damage variables.

Based on the data in Table 5, the damage variable variation law is shown in Table 6. The relationships of the damage variables with the confining pressure and the number of cycles were plotted according to Table 6 (Fig. 15).

**Table 6:** Statistical table of damage variables (Energy unit: kJ/m^3^).

Confining pressure/MPa	Cycle 1	Cycle 2	Cycle 3	Cycle 4	Cyclic loading and unloading section			Unloading section			D/D′ (%)
	D1	D2	D3	D4	Ud	U	D	Ud′	U′	D′	
13.0	0.349	0.316	0.298	0.311	449.30	1828.28	0.246	254.19	591.35	0.430	57.17
13.5	0.286	0.260	0.244	0.245	345.99	1723.63	0.201	182.50	531.18	0.344	58.42
14.0	0.260	0.230	0.213	0.202	253.39	1561.67	0.162	160.94	487.11	0.330	49.11
14.5	0.257	0.226	0.209	0.198	244.38	1546.71	0.158	158.30	485.62	0.326	48.47
15.0	0.253	0.224	0.208	0.197	240.02	1524.24	0.157	155.05	480.73	0.323	48.82
17.0	0.250	0.220	0.202	0.192	218.17	1451.17	0.150	148.55	463.57	0.320	46.92
20.0	0.253	0.221	0.202	0.190	195.34	1351.87	0.144	146.57	445.32	0.329	43.90

It can be seen from Table 6 and Fig. 15 that

1. The number of cycles exerts an inhibitory effect on damage variables at the beginning of the cycle and shows a facilitating effect at the end of the cycle, which is similar to its effect on the dissipated energy density. Damage variables reach the minimum value in the 3rd cycle at the unloading quantity of 86.67% and 93.33%, which is consistent with the previous results. They first decrease from 0.286 and 0.349 to 0.244 and 0.298 with a reduction of 0.042 and 0.051 and then increase to 0.245 and 0.311 with a rise of 0.001 and 0.013, respectively. For unloading quantity less than 80.00%, the damage variable decreases on average from 0.255 to 0.196, a drop of 0.059. It can be predicted that the damage variable in the 5th cycle should be greater than that in the 4th cycle.
2. The differences in damage variables within each hysteresis loop are small but are large under different unloading quantities (confining pressure). During the increase of the unloading quantity from 0 to 93.33% (the decrease of confining pressure from 20.0 to 13.0 MPa), the damage variable changes abruptly at 80.00% unloading quantity (confining pressure of 14.0 MPa). The value increases slowly from 0.217 to 0.226 and then steeply to 0.319, with an increment of 0.009 and 0.093 in the two stages, respectively.
3. Overall, the damage variables (unloading section and cyclic loading and unloading section) exhibit a significant nonlinear relationship with the confining pressure. They develop steadily when the unloading quantity is small and significantly increase when it is large, indicating that the damage level inside the rock is higher when the unloading quantity is larger.The damage variables in the cyclic loading and unloading stage are smaller than those in the unloading section at the same confining pressure. The former is about 43.90–58.42% of the latter, indicating that although both the unloading process and the cyclic loading and unloading process damage specimens, the unloading process causes greater energy dissipation and a higher degree of damage, playing a dominant role in specimen failure.

### Microstructure analysis

To analyze the effects of the unloading process and the cyclic loading and unloading process on the microstructure of sandstone, typical SEM photographs of rock samples in Option 1, Option 2 and Option 3 are shown in Table 7.

**Table 7:** Characteristics of the microstructure of sandstone samples under three options.

Test option	SEM photos of typical rock samples		Characteristic
Option 1	Confining pressure of 12.5 MPa	Confining pressure of 20.0 MPa	The mineral grains are tightly structured, the cement is dense, and there are some tiny cracks
Option 2	Destruction under 12.5 MPa confining pressure	Destruction under 12.5 MPa confining pressure	Mineral grains begin to break up and rock chips are attached to the surface. The cement is loose, and cracks are gradually developed
Option 3	Destruction under 13.0 MPa confining pressure	Destruction under 20.0 MPa confining pressure	The edges of mineral grains tend to be smooth, and some mineral grains are broken. More rock chips are attached, and cracks are gradually penetrated

As can be seen in Table 7, the microfabrication characteristics of the rock samples vary significantly:

1. After the specimen test failure, the internal microcracks increase, and the cracks can be divided into: intergranular cracks, intracrystalline cracks and transgranular cracks^35,36^. In the conventional triaxial compression test, the structure of mineral grains inside the rock sample is relatively dense, and there are microfine cracks (intergranular cracks) at the boundary of a few mineral grains; the ports are relatively straight, and some sections are scattered with rock debris; the cementation between the grains is good, and internal cracks are not developed. After unloading, intergranular cracks develop obviously, and cracks between the grains gradually expand and connect; some cracks develop along the crystal surface (intracrystalline cracks), and rock debris increases significantly. After cycling and unloading, due to the interaction of the slip surface, the angles of the mineral grains tend to be rounded and smooth; cracks are gradually connected and enlarged, mainly along the intergranular cracks and accompanied by some cracks through the grain (transgranular cracks); local mineral grains flake off, and the structure of the rock sample gradually becomes loose.
2. When the number of cycles is small, the rock debris generated by fracture damage due to unloading will fill into the nearby fractures, which contributes to a denser contact state^34^, improving interparticle cementation strength and overall deformation characteristics (similar conclusions can be drawn from the previous studies). As the number of cycles increases, more debris is generated on the surface due to interfacial friction. The microcracks expand along the weak surface, and closed cracks reopen to develop, accompanied by the generation of new cracks. The cumulative damage is larger, especially under high unloading quantity, which provides more reaction space, and the compacted cracks and secondary cracks are more easily activated. The cyclic loading and unloading process is relatively fast, and the mechanical and deformation characteristics of the rock samples deteriorate fast accordingly.

## Conclusions

In this paper, the effects of the cyclic loading and unloading process and the unloading process on the mechanical properties of sandstone were investigated by conventional triaxial compression tests, triaxial compression tests on unloading damaged specimens, and cyclic loading and unloading tests on unloading damaged specimens. Conclusions were obtained as follows.

1. The sandstone shows brittle failure under the three test options. The unloading process can reduce the peak strength of sandstone. As the unloading quantity increases, the strength decay becomes significant (more significant when the unloading quantity exceeds 80.00%). The cyclic loading and unloading process can further weaken the load-bearing capacity of sandstone, but the impact is small. The cyclic loading and unloading process can cause greater deformation of sandstone (positively correlated with unloading quantity).
2. The dominant failure mode of sandstone under the three test options is shear failure. With the increase in unloading quantity, the failure mode of the sandstone is gradually transformed from compression-shear failure to tensile-shear failure. After superimposing cyclic loading and unloading, transverse fracture perpendicular to the direction of cyclic load action is formed in sandstone, and the specimens are more prone to fracture failure.
3. The dissipated energy density during cyclic loading and unloading is significantly smaller than that during unloading, and shows a decreasing trend at the early stage of cycling, which indicates that the cyclic action at the early stage is beneficial to crack closure. The later cyclic action accelerates crack expansion (positively correlated with unloading quantity) and specimen failure.
4. The damage variables during cyclic loading and unloading are about 50.00% of those during unloading, indicating that both the unloading and the cyclic loading and unloading can exacerbate the damage inside the specimen. The unloading process plays a decisive role in the development of cracks inside the specimen and is the dominant factor for the failure of the specimen.
5. The extension of microcracks within the sandstone is dominated by intergranular cracks. The unloading process promotes the generation of a small number of intracrystalline cracks, and the number of cracks increases with the increasing unloading quantity. After the cyclic loading and unloading process, transgranular cracks are formed, the grains become smoother, and the structure becomes looser.

## Data Availability

The datasets used and/or analyzed during the current study are available from the corresponding author on reasonable request.
